# Resistance Exercise, Electrical Muscle Stimulation, and Whole-Body Vibration in Older Adults: Systematic Review and Meta-Analysis of Randomized Controlled Trials

**DOI:** 10.3390/jcm9092902

**Published:** 2020-09-08

**Authors:** Nejc Šarabon, Žiga Kozinc, Stefan Löfler, Christian Hofer

**Affiliations:** 1 Faculty of Health Sciences, University of Primorska, Polje 42, SI-6310 Izola, Slovenia; ziga.kozinc@fvz.upr.si; 2 InnoRenew CoE, Human Health Department, Livade 6, SI6310 Izola, Slovenia; 3 S2P, Science to practice, Ltd., Laboratory for Motor Control and Motor Behavior, Tehnološki Park 19, SI-1000 Ljubljana, Slovenia; 4 Andrej Marušič Institute, University of Primorska, Muzejski Trg 2, SI-6000 Koper, Slovenia; 5 Physiko- & Rheumatherapie, Institute for Physical Medicine and Rehabilitation, 3100 St. Pölten, Austria; stefan.loefler@rehabilitationresearch.eu; 6 Centre of Active Ageing—Competence Centre for Health, Prevention and Active Ageing, 3100 St. Pölten, Austria; 7 Ludwig Boltzmann Institute for Rehabilitation Research, 3100 St. Pölten, Austria; christian.hofer@rehabilitationresearch.eu

**Keywords:** sarcopenia, falls, elderly, resistance exercise, vibration, electrical stimulation

## Abstract

It has been shown that resistance exercise (RT) is one of the most effective approaches to counteract the physical and functional changes associated with aging. This systematic review with meta-analysis compared the effects of RT, whole-body vibration (WBV), and electrical muscle stimulation (EMS) on muscle strength, body composition, and functional performance in older adults. A thorough literature review was conducted, and the analyses were limited to randomized controlled trials. In total, 63 studies were included in the meta-analysis (48 RT, 11 WBV, and 4 EMS). The results showed that RT and WBV are comparably effective for improving muscle strength, while the effects of EMS remains debated. RT interventions also improved some outcome measures related to functional performance, as well as the cross-sectional area of the quadriceps. Muscle mass was not significantly affected by RT. A limitation of the review is the smaller number of WBV and particularly EMS studies. For this reason, the effects of WBV and EMS could not be comprehensively compared to the effect of RT for all outcome measures. For the moment, RT or combinations of RT and WBV or EMS, is probably the most reliable way to improve muscle strength and functional performance, while the best approach to increase muscle mass in older adults remains open to further studies.

## 1. Introduction

With rising life expectancy and the increasing proportion of older adults in the population [1,2], effective interventions that promote lifelong well-being and health are more needed than ever before. There is no doubt that performing physical exercise is one of the most effective ways for older adults to maintain functional independence, maintain physical abilities, and reduce the risk of various diseases and injuries [3,4,5,6,7]. One of the most notable changes associated with aging is sarcopenia, which is characterized by a loss of muscle mass and other subsequent changes, such as reduced muscle strength and impaired functional ability [8]. Together with nutritional interventions, resistance exercise training (RT) seems to be the most effective approach to prevent and treat sarcopenia [9,10,11]. Falls are also one of the major problems in the older adult population [12] and are thus given considerable attention in terms of prevention. It has been shown that the best way to prevent falls is by performing RT alone or in combination with other exercise types or other interventions [13,14]. Despite extensive research regarding the effects of resistance exercise on sarcopenia, fall risk, and general health of older adults, the recommendations for prescribing exercises in this population are still relatively vague and generic [3,11,15]. In contrast, previous studies have investigated several factors that are worth considering in order to maximize the effects of RT for older adults, such as intensity [16], speed of movement [17], and supervision of the training sessions [18]. Certain types of RT, such as speed-power training [19], are also increasingly being investigated as potentially superior to traditional resistance exercise.

Recent literature reviews have found numerous barriers, such as decreased physical ability, walking disability, lack of companionship, and lack of motivation, that are decreasing the participation of older adults in exercise programs [20,21]. For this reason, different methods to combat sarcopenia, prevent falls, and increase well-being in older adults should be considered as an alternative to RT. Recently, whole-body vibration (WBV) has been shown to improve postural balance [22] and muscle strength [23] and to reduce the likelihood of falls in older adults [24]. WBV is therefore a possible alternative to RT; however, direct comparisons between the effects of RT and WBV are lacking. Roelants et al., reported similar improvements in knee extension strength, jumping performance, and speed of movement after 12 and 24 weeks of RT and WBV interventions in older women [25]. Similarly, Bogaerts et al., showed comparable effects of WBV and RT on muscle mass and muscle strength in older men [26]. Another promising alternative to RT is electrical muscle stimulation (EMS) [27,28,29,30,31]. EMS has been shown to improve functional performance of aging muscles [27,31] and to counteract muscle decline in old age [30]. Moreover, EMS has been appreciated as a convenient intervention for older adults with lower physical abilities or low motivation to exercise [32].

Although many positive effects of RT, WBV, and EMS in older adults have been consistently demonstrated, it is not entirely clear which interventions should be prioritized for the best health benefits. Moreover, studies often follow only a limited set of outcome measures, making comparisons between interventions difficult. Therefore, the objective of this work was to provide a comprehensive systematic review with meta-analysis of high-quality studies that assessed the effects of RT, WBV, or ES in older adults. To obtain a broad overview of these effects, we included studies that assessed various outcome measures, including muscle strength, body composition, and muscle morphology, and the outcomes of functional performance tests. In addition, the aim of this review was to examine the effects of several independent variables, pertaining to the intervention programs, such as (but not limited to) intervention duration, weekly frequency, volume, intensity, supervision, and compliance. We hypothesized that RT, WBV, and EMS will have similar effects on body composition, muscle strength, and functional performance.

## 2. Materials and Methods

### 2.1. Inclusion Criteria

Study inclusion criteria were structured according to PICOS tool [33]:

- Population (P): Male or female older adults. The criterion for inclusion was mean sample age ≥ 65.0 years. Patients with sarcopenia were included if they met this criterion (age ≥ 65.0 years); however, sarcopenia was not an inclusion criterion.
- Intervention (I): RT, EMS, or WBW interventional programs of any duration. Studies exploring multimodal interventional programs (e.g., RT programs combined with stretching exercise) were excluded.
- Comparisons (C): Control groups, receiving no intervention or placebo intervention. Groups that received cognitive training or other non-physical interventions were also accepted as control groups. Studies in which control groups received any type of exercise, vibration intervention, electrical stimulation, or nutritional supplementation were excluded.
- Outcomes (O): (a) Muscular strength or power, not limited to type of testing or body part; (b) body composition and muscle architecture (including body fat, fat free mass, muscle mass, regional muscle mass, skeletal muscle mass, cross-sectional muscle area, circumference measures, and sarcopenia index) and (c) functional mobility outcomes (timed up-and-go test, stepping tests, sit-stand tests, functional reach tests, etc.).
- Study design (S): Only randomized controlled trials (RCT) that included at least one intervention group (RT, EMS, or WBV) and control group.

### 2.2. Search Strategy

Multiple databases of scientific literature (PubMed, Cochrane Central Register of Controlled Trials, PEDro, and ScienceDirect) were searched in May 2020 without regard to the date of publication. For the databases that enable using Boolean search operators, we used the following combination of search key words: (sarcopenia OR muscle atrophy OR muscle wasting) and (training OR exercise OR vibration OR electrical stimulation OR electrostimulation OR magnetic stimulation OR vibration training OR physical therapy) and (strength OR power OR muscle mass OR muscle diameter) and (elderly OR older OR older adults OR ageing OR age-related). Otherwise, we used several reduced combinations of key words, including, but not limited to resistance exercise older adults, vibration training elderly, electrical stimulation elderly and older adults sarcopenia intervention. Additionally, reference lists of several review articles describing interventions for older adults were carefully scrutinized. Finally, we carefully reviewed reference lists of all articles that were already retrieved through the database search and were published within the last 4 years. The database search was performed independently by two authors (N.Š. and Ž.K.). Two reviewers (N.Š. and S.L.) also screened the titles and the abstracts independently. Potentially relevant articles were screened in full text, followed by additional screening for their eligibility by the additional reviewers.

### 2.3. Data Extraction

The data extraction was carried out independently by two authors (Ž.K. and C.H.) and disagreements were resolved through consultation with other authors. The extracted data included: (a) baseline and post-intervention means and standard deviations for all eligible outcome measures for interventional and control groups; (b) baseline demographics of participants (gender, age, body height, body mass, body mass index); (c) intervention characteristics (target body area (upper, lower or whole-body), duration of the intervention, number of sessions per week, volume (number of exercises, sets, and repetitions), breaks between exercises and sets, supervision, and progression of exercise difficulty). For studies examining RT, we also extracted the type of load used (bodyweight, machine, elastics, weights, etc.) and intensity as a percentage of 1-maximum repetition (1RM) or subjective measures, such as the Borg scale. For EMS studies, we further extracted the stimulation frequency and amplitude, the stimulated body parts, pulse shapes, and breaks between repetitions or sets. For WBV studies, we additionally extracted the amplitude and the frequency that was used during training. Data were carefully entered into Microsoft Excel 2016 (Microsoft, Redmond, WA, USA). If the data were presented in a graphical rather than tabular form, we used Adobe Illustrator Software (version CS5, Adobe Inc., San Jose, CA, USA) to accurately determine the means and standard deviations. In case of missing data, the corresponding author of the respective articles was contacted by e-mail. If no response was received after 21 days, the author was contacted again. If the author did not reply to the second inquiry, the data was considered irretrievable.

### 2.4. Assessment of Study Quality

Two authors (Ž.K. and N.Š.) evaluated the quality of the studies using the PEDro tool [34], which assesses study quality based on a ten-level scale. Potential disagreements between ratings were resolved by consulting the other authors. Studies scoring from 9–10 were considered as “excellent,” 6–8 as “good,” 4–5 as “fair,” and less than 4 as “poor” quality. The PEDro scale was chosen because it was developed specifically to assess the quality of randomized controlled trial studies evaluating physical therapist interventions [34].

### 2.5. Data Analysis and Synthesis

The main data analyses were carried out in Review Manager (Version 5.3, Copenhagen: The Nordic Cochrane Centre, The Cochrane Collaboration, 2014, London, UK). Before the results were entered into the meta-analytical model, the pre-post differences and pooled standard deviations were calculated according to the following formula SD = √[(SD_pre_^2^ + SD_post_^2^) − (2 × r × SD_pre_ × SD_post_). The correction value (r), which represents the pre-test–post-test correlation of outcome measures, was conservatively set at 0.75. It should be noted that a change in the correction value in the range between 0.5 and 0.9 had little effect on the pooled SD and would not change the outcomes of the meta-analyses. For the meta-analyses, the inverse variance method for continuous outcomes with a random-effects model was used. The pooled effect sizes were expressed as mean difference (MD) where possible, which allows the effect size to be expressed in units of measurement. Where this was not possible due to the heterogeneity of the outcome variables (e.g., muscle strength reported in kg, N, Nm, N/kg, and Nm/kg), the effect sizes were expressed as standardized mean difference (SMD). For MD and SMD, the respective 95% confidence intervals were also calculated and reported.

Basic analysis compared the effects of the RT, EMS, and WBV interventions. Further subgroup analyses were conducted where possible (depending on the number of studies reporting a given outcome) based on several independent variables, related to the characteristics of the interventions (e.g., weekly number of sessions). Some outcomes did not appear in EMS and WBV studies and were thereby only analyzed in view of RT studies. Statistical heterogeneity among studies was determined by calculating the I2 statistics. According to Cochrane guidelines, the I2 statistics of 0% to 40% might not be important, 30% to 60% may represent moderate heterogeneity, 50% to 90% may represent substantial heterogeneity, and 75% to 100% indicates considerable heterogeneity [35]. The threshold for statistical significance was set at *p* ≤ 0.05 for the main effect size and the subgroup difference tests.

Sensitivity analysis was performed when deemed necessary i.e., by examining the effect of exclusion of certain studies from the analyses (e.g., studies that could have included subsets of previous studies, studies with very low compliance, studies that did not report intensity, studies with and without elderly with sarcopenia, etc.). The sensitivity analyses showed no or very little effect on the main results (SMD changes = 0.01–0.10), except where noted and reported in the results.

## 3. Results

### 3.1. Summary of Search Results

**Figure 1:**
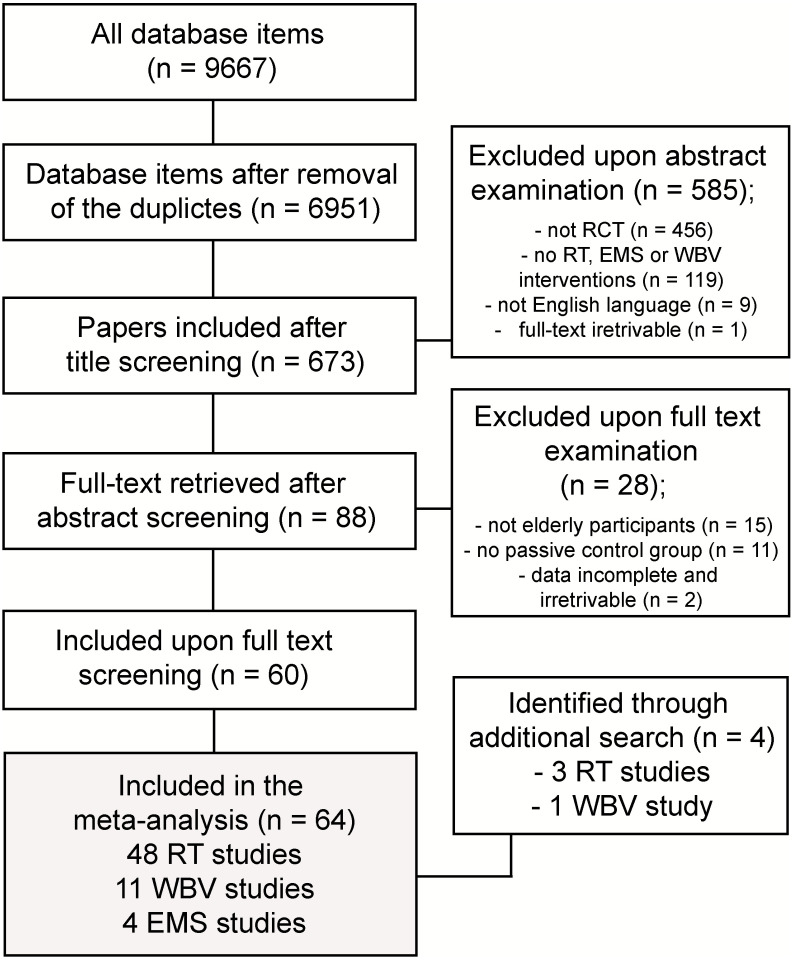
Summary of search results. RT—resistance training; WBV—whole-body vibration; EMS—electrical muscle stimulation.

The results of the search steps are summarized in Figure 1. The search resulted in 64 studies in total, 48 of which included RT interventions (55 intervention groups in total), 12 included WBV interventions (14 intervention groups in total) and 4 included EMS interventions (4 intervention groups in total). A table encompassing all the details regarding the participants, interventions and outcomes of individual studies is included in Supplementary data 1.

### 3.2. Study Quality Assessment

The PEDro scale scores indicated overall fair to good quality of the RT studies (mean = 5.25 ± 1.26; median = 5.0; range = 2–8) and WBV studies (mean = 5.41 ± 1.24; median = 5.5; range = 4–7). Studies exploring EMS were all rated as good (mean = 6.52 ± 1.03; median = 6.0; range = 6–8). The most common items that almost all studies failed to satisfy were blinding of the subjects, therapists and assessors.

### 3.3. Participant Data and Intervention Characteristics

In total, there were 2017 participants (1158 in intervention groups and 1026 in control groups) in the RT studies, 606 in WBV studies (325 in intervention groups and 284 in control groups), and 192 in the EMS studies (99 in intervention groups and 93 in control groups). Across all studies, the pooled participant age was 73.5 ± 4.8 years (range of means: 65–92 years), the pooled participant body mass was 65.8 ± 10.33 kg (range of means: 40.5–101.8 kg), and the pooled body mass index was 26.39 ± 3.77 kg/m^2^ (range of means: 18.8–36.7 kg/m^2^). In total, 36 included participants of both genders, 24 studies included only females, and 4 studies included only males. In 16 RT studies, sarcopenia was listed as an inclusion criterion. In 47 studies, the interventions were supervised, while the interventions in the remaining studies were not supervised (*n* = 9) or the information regarding the supervision was missing (*n* = 7). The most typical duration of the interventions was 12 weeks (*n* = 28), while 12 interventions were shorter (4 interventions lasted 5–6 weeks, and 8 interventions lasted 8–11 weeks) and 23 interventions were longer (12 interventions lasted 13–24 weeks, and 11 interventions lasted 25 weeks or more). Most interventions included either 2 (*n* = 23) or 3 (*n* = 32) sessions per week, while 5 interventions were performed once per week and 3 interventions were performed 4–5 times per week. Only 4 WBV and 19 RT studies reported adherence to the intervention program, with mean values of 90 ± 3% and 84 ± 9%, respectively.

Across the RT studies, 14 intervention programs used machines, 6 used free weights, 5 used elastic resistance, 4 implemented bodyweight exercises, 1 used weighted tai-chi exercises, and 1 used isoinertial exercises on a flywheel device. The remaining 17 studies used mixed approaches (5 combined bodyweight and elastic exercise, 2 combined free weights and bodyweight exercises, 3 combined free weights and machines, and 7 used more three or four types of load). RT interventions included either full body workout (*n* = 32) or focused on the lower limb muscles (*n* = 16), while no interventions focused only on the trunk or the upper limbs. Most often (*n* = 29), the intervention included a combination of single-joint and multi-joint exercises; however, some interventions included predominantly single-joint (*n* = 12) or multi-joint (*n* = 7) exercises. The volume of exercise varied substantially between studies, with the number of exercises ranging from 1 to 12 (mean: 5.9 ± 2.9), the number of sets ranging from 1 to 5 (mean: 2.7 ± 0.8), and number of repetitions within sets ranging from 7 to 25 (mean: 11.0 ± 3.5). Intensity was set as percentage of 1-repetition-maximum in 27 studies (mean: 66.2 ± 15.3%; range: 20–80%) or using the 6–20 Borg scale for assessment of the rate of perceived exertion in 10 studies (all studies used 13 as the target value). One study determined the intensity as percentage of maximal heart rate (set between 60 and 80%). The remaining 12 studies did not report the intensity of the exercise. Breaks between sets were reported in 11 studies and ranged from 30 s to 150 s (mean: 100 ± 45 s). Breaks between exercises were only reported in 5 studies (range: 90–180 s).

In WBV studies, the number of exercises ranged from 1 to 9 (mean: 3.8 ± 3.1) and the number of sets ranged from 1 to 10 (mean: 3.5 ± 2.7). With the exception of 1 study, which used highly varying vibration frequency (27–114 Hz), the frequencies used ranged from 20 to 60 Hz (mean: 35.7 ± 10.1 Hz). The amplitude of the vibration ranged from 2 to 6 mm (mean: 3.8 ± 1.4 mm). Breaks between sets ranged from 30 to 180 s (mean: 75 ± 53.8 s).

Finally, 3 EMS studies targeted full body (all used stimulation frequency of 85 Hz, impulse width at 350 μs, moderate intensity (subjectively determined) and lasted 20 min per session), while 1 study stimulated only the lower limbs (frequency: 100 Hz; amplitude: 40–120 mA; impulse width: 400 μs).

### 3.4. Effects of RT and WBV on Muscle Strength

**Figure 2:**
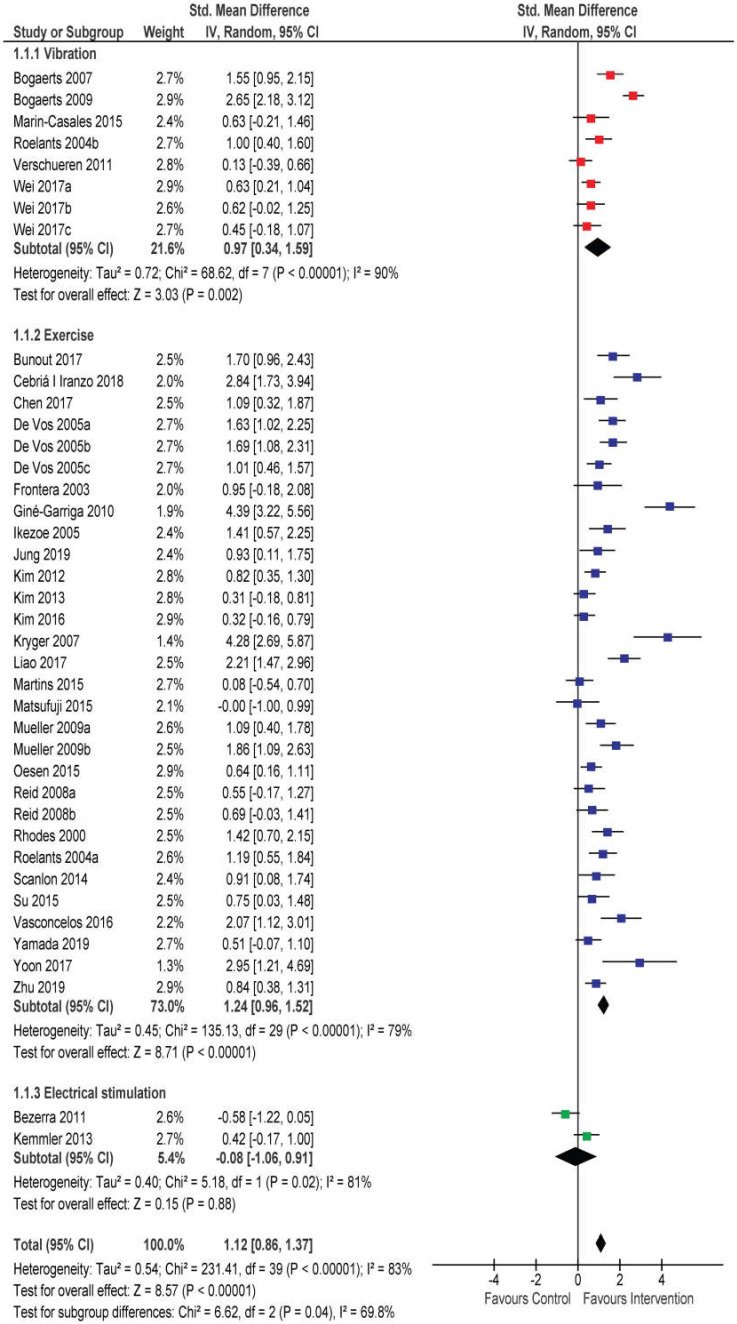
Effects of whole-body vibration, resistance exercise, and electrical muscle stimulation interventions on knee extension strength.

Knee extension strength was by far the most common outcome across studies and was reported in 2 EMS studies with 2 intervention groups [36,37], 6 WBW studies with 8 intervention groups [25,26,38,39,40,41,42], and 26 RT studies with 29 intervention groups [25,43,44,45,46,47,48,49,50,51,52,53,54,55,56,57,58,59,60,61,62,63,64,65,66,67]. In total, 5 studies measured isokinetic strength (1 study at 30°/s, 3 studies at 60°/s and 1 study sat 90°/s), and the rest measured isometric strength. Figure 2 displays the main analysis, comparing the effect of WBV, RT, and EMS on knee strength. Due to substantial discrepancy between the studies in terms of units of reporting, only the SMD could be computed.

There was a statistically significant increase in knee extension strength in the intervention groups across all studies compared to control groups (SMD = 1.12 (0.86–1.37); *p* < 0.001; I^2^ = 83%). Both WBV interventions (SMD = 0.97 (0.34–1.59); *p* = 0.00; I^2^ = 90%) and RT interventions (SMD = 1.24 (0.96–1.52); *p* < 0.001; I^2^ =79%) improved knee extension strength, while EMS did not (SMD = −0.08 (−1.08–0.91); *p* = 0.88; I^2^ = 81%). RT appeared superior to WBV; however, the difference between intervention types was not statistically significant (*p* = 0.32). For WBV, the subgroup analysis was performed for intervention duration and indicated that interventions longer than 24 weeks have a higher effect (SMD = 1.61 (0.35–2.87) than interventions lasting up to 12 weeks (SMD = 0.55 (0.21–0.88) or interventions lasting 13–24 weeks (SMD = 0.55 (−0.29–1.40)), although the subgroup test showed that this difference was not statistically significant (*p* = 0.28). Within the RT studies, most interventions lasted 12 weeks (17/26 studies). Subgroup analyses showed no effect of intervention duration on knee strength increases (SMD = 0.94–1.26 across subgroups). The effect of RT was the highest in studies with participants aged > 80 years (SMD = 1.76 (1.01–2.52), lower in the < 70-year-old subgroup (SMD = 1.17 (0.73–1.61) and the lowest in the 70–80-year-old subgroup (SMD = 0.95 (0.65–1.25)) (*p* = 0.14 for subgroup differences). The effect was comparable in studies using predominantly single-joint (SMD = 1.38 (0.70–2.07), predominantly multi-joint (SMD = 1.12 (0.33–1.90)), or a combination of single- and multi-joint exercises (SMD = 1.27 (0.91–1.62)) (*p* = 0.88 for subgroup differences). No differences between studies were found (*p* = 0.68) based on the type of resistance, though there was a trend for higher effect of interventions based on machine training (SMD = 1.36 (0.97–1.75)) and free weights (SMD = 1.33 (0.37–2.29)) compared to elastic resistance (SMD = 0.91 (0.20–1.63)) and approaches that combined multiple types of resistance (SMD = 0.98 (0.49–1.47)). Finally, studies were grouped according to number of sessions per week and no differences were found between interventions performed ≤2 times per week (SMD = 1.30 (0.92–1.68)) and ≥3 times per week (SMD = 1.15 (0.75–1.55)) (*p* = 0.59 for subgroup differences).

Sensitivity analysis was performed to examine the effect of certain concerns regarding the studies. Since it was not entirely clear if Bogaerts et al. (2007 and 2009, see Figure 2, top section) reported the data for entirely different sample in the two studies, we excluded the study with smaller sample size. The pooled effect of WBV was decreased from 0.97 to 0.88; however, it was still statistically significant (*p* = 0.01). Furthermore, 4 WBV studies included in this analysis involved some component (lunges, squats) of RT. Therefore, it is unclear if this RT component contributed to the overall improvements. Removing these studies from the analysis yields a lower overall effect (SMD = 0.59 (0.30–0.87), which is statistically still significant (*p* = 0.03); however, with this reduction in studies, the subgroup analyses indicate statistically significant difference (*p* = 0.001) between RT and WBV, indicating the superiority of RT compared to WBV without any RT components. Additionally, we repeated the analysis with exclusion of RT studies on sarcopenia patients (SMD increased from 1.24 to 1.34) and vice versa (SMD dropped to 1.01). Therefore, a slight tendency for larger effect in healthy older adults was indicated. A final sensitivity analysis was performed for type of measurement. Removing the studies that measured isokinetic strength increased the main effect slightly (from 1.24 to 1.33). However, the studies with isokinetic measurements also had large and statistically significant pooled effect (SMD = 0.88; *p* < 0.001), which suggest isokinetic and isometric strength both substantially increased with RT.

**Figure 3:**
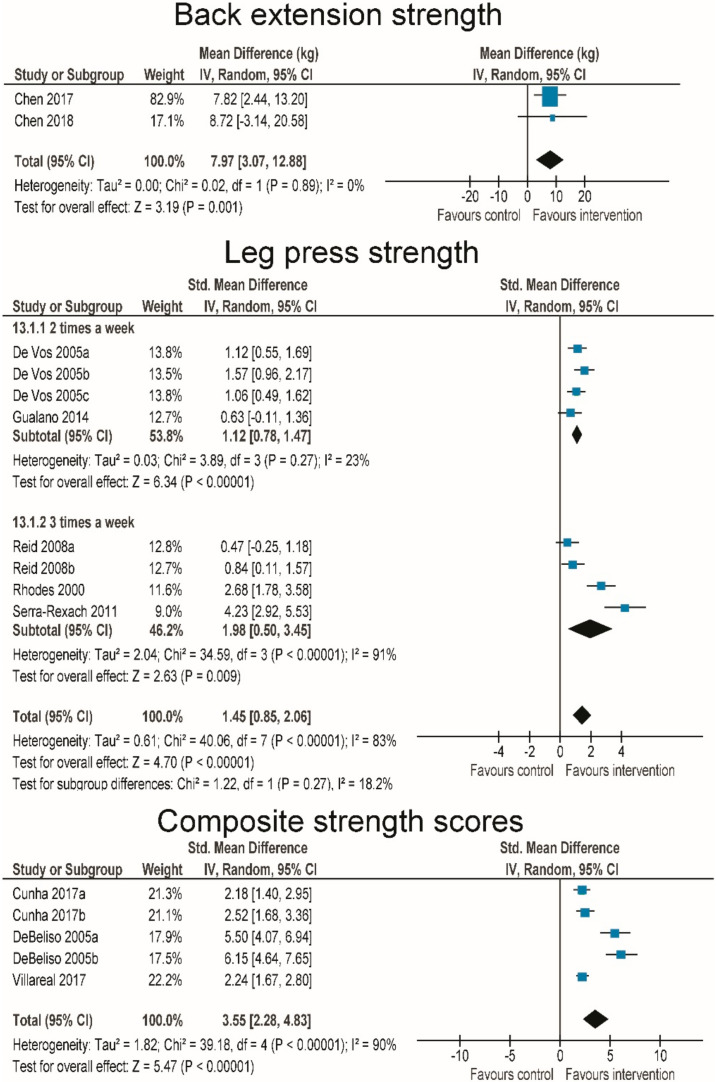
Effect of resistance exercise interventions on back extension, leg press, and composite strength scores.

Leg press strength was reported in 5 RT studies (8 interventional groups) [46,60,61,68,69]. There was a statistically significant increase in intervention groups across studies (SMD = 1.45 (0.85–2.06); *p* < 0.001; I^2^ = 83%) (Figure 3). Interventions performed 3 times per week tended to have a larger effect (SMD = 1.98 (0.50–3.45)) than interventions performed 2 times per week (SMD = 1.12 (0.78–1.47)), but the subgroup difference was not statistically significant (*p* = 0.27 for subgroup differences). Two RT studies reported back extensor strength [45,70] and showed a statistically significant increase (MD = 7.97 kg (3.07–12.88 kg); *p* < 0.001; I^2^ = 0.0%) (Figure 3). Three RT studies [71,72,73] reported a composite score for strength (i.e., sum of several strength tasks). There was a statistically significant improvement in intervention groups (SMD = 3.55 (2.28–4.83); *p* < 0.001; I^2^ = 90%) (Figure 3). Grip strength was reported in 19 RT studies [44,45,49,52,53,55,56,59,61,65,67,69,70,74,75,76,77,78,79]. There was a mean increase of 1.48 kg (0.26–2–23 kg; *p* < 0.001) across studies with pre-post mean differences ranging from −1.00 to 5.70 kg.

### 3.5. Effects of RT on Body Composition

**Figure 4:**
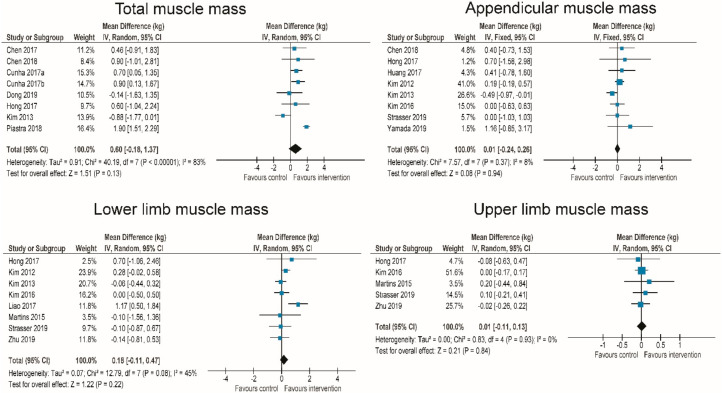
Effect of resistance exercise interventions on muscle mass.

Muscle mass was reported in 7 RT studies (8 intervention groups) [45,52,70,71,74,78,80]. Compared to control groups, there was not a statistically significant increase in muscles mass in intervention groups across studies (MD = 0.60 kg (−0.18–1.37 kg); *p* = 0.13; I^2^ = 83%) (Figure 4). There were no differences between interventions performed 2 times a week (MD = 0.60 kg (−1.01–2.22 kg)) and 3 times a week (MD = 0.68 kg (0.23–1.14 kg)) (*p* = 0.93 for subgroup differences). Appendicular muscle mass was reported in 7 RT studies [51,52,53,65,70,80,81,82]. The pooled effect showed no change after RT interventions compared to control groups (MD = 0.01 kg (−0.26–0.28 kg); *p* = 0.92; I^2^ = 8%) (Figure 4). Lower-limb muscle mass was reported in 8 RT studies [51,52,53,55,56,67,80,82], with an overall small and statistically non-significant increase (MD = 0.18 kg (−0.11—0.47 kg); *p* = 0.22; I^2^ = 45%) (Figure 4). No statistically significant differences were shown between interventions performed 3 times per week (MD = 0.55 kg (−0.44–1.55 kg)) compared to interventions performed 2 times per week (MD = 0.10 kg (−0.10–0.31 kg)) (*p* = 0.39 for subgroup differences). Upper limb muscle mass was reported in 5 RT studies [53,56,67,80,82], and the pooled effect was negligible (MD = 0.01 kg (−0.11–0.13 kg); *p* = 0.84; I^2^ = 0%) (Figure 4).

Fat-free mass was recorded in 2 WBV [39,83], 7 RT [55,62,73,74,75,76,81], and 1 EMS studies [84], with a very small and statistically non-significant reduction across studies (MD = −0.27 kg (−0.84–0.31 kg); *p* = 0.46; I^2^ = 0%). The pooled effect of the two WBV studies showed a slight increase (MD = 0.53 kg (−1.75–2.81 kg); *p* = 0.15), as did one EMS study (MD = 0.61 kg (−0.81–2.03 kg); *p* = 0.40), while there was a small and statistically non-significant decrease across RT interventions (MD = −0.60 kg (−1.28–0.09 kg); *p* = 0.09). The differences between WBV, RT, and EMS were not statistically significant (*p* = 0.25).

Body fat mass was reported in 2 WBV [39,41] and 14 RT (16 intervention groups) studies [45,50,53,55,58,62,70,71,73,74,76,80,81,85], with a statistically significant decrease overall (SMD = −0.65 (−1.09–−0.21); *p* < 0.001; I^2^ = 86%). For the purposes of MD calculation, three studies (4 intervention groups) [45,58,74] that reported body mass in kg instead of the percentage of body weight were removed and the analysis was repeated. SMD slightly increased (SMD = −0.74) and MD calculation showed a mean reduction of body fat mass percentage of −1.99% (−3.75–−0.22%).

**Figure 5:**
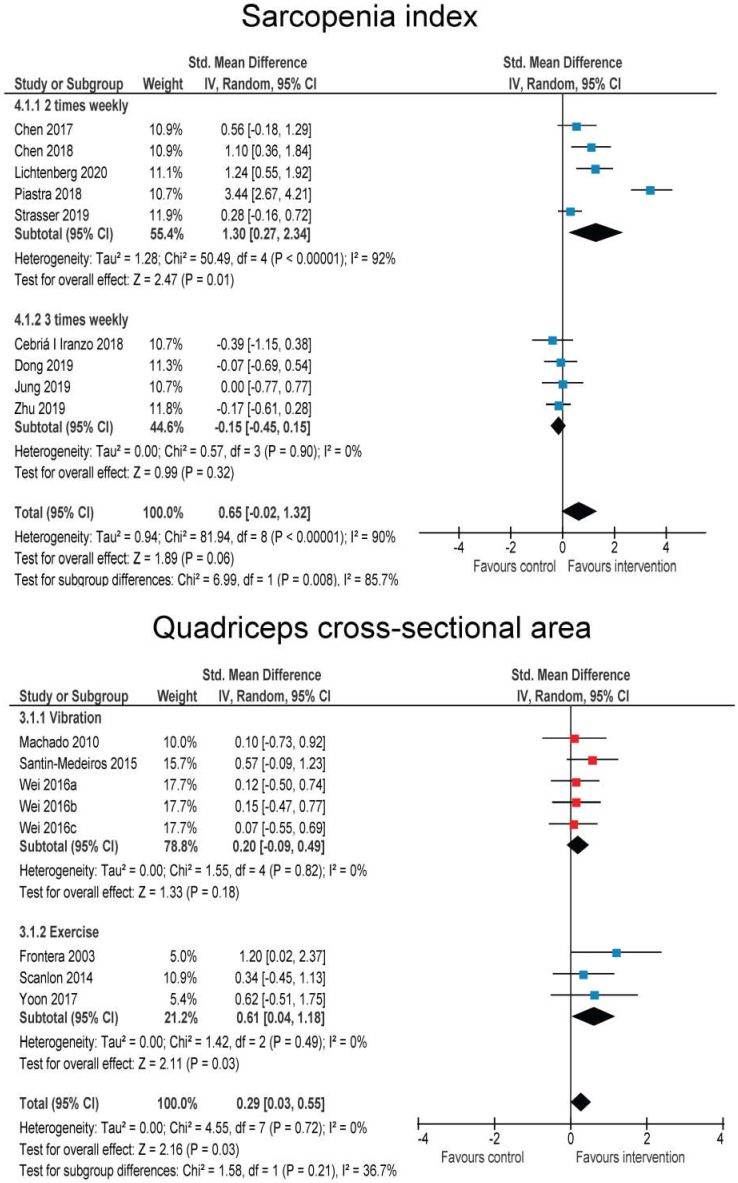
Effects of whole-body vibration and resistance exercise on sarcopenia index and quadriceps cross-sectional area.

Nine RT studies [44,50,67,70,74,77,78,82] and one EMS study [86] also reported the sarcopenia index (sometimes termed skeletal muscle index) (Figure 5). Mainly (7 studies), the index was computed as the ratio of appendicular skeletal muscle mass and the square body height. However, since two studies did not report the exact calculation of the index, we opted for SMD in order analyses to be conservative. There was a moderate, but statistically non-significant improvement across all studies (SMD = 0.65 (−0.02–1.32); *p* = 0.06; I^2^ = 90%). Subgroup analyses favored RT interventions, performed 2 times weekly (*p* = 0.008); this is being heavily influenced by one RT study that showed substantial improvement (SMD = 3.44). Most of the studies showed very small negative or very small positive effects, while the pooled effect was heavily influenced by the aforementioned study. Furthermore, 3 WBV studies [42,87,88] (5 intervention groups) and 3 RT studies [47,62,66] reported the quadriceps muscle (or individual heads of quadriceps muscle) cross-sectional area. In order to obtain a sufficient number of studies for meaningful comparison, these results were compared together and expressed as SMD. Overall, there was a statistically significant effect of interventions (SMD = 0.29 (0.03–0.55); *p* = 0.03; I^2^ = 0%) (Figure 5). Subgroup differences showed no differences between RT (SMD = 0.61 (0.04–1.18)) and WBV (SMD = 0.20 (−0.09–0.49) (*p* = 0.21 for subgroup differences). For the RT studies (all reported the cross-sectional area for the full quadriceps muscle), the MD was 1.80 (0.51–3.09) cm^2^. One RT study [57] reported thigh circumference, with no effect of the intervention (MD = −0.10 cm (−2.55–2.35 cm); *p* = 0.94; I^2^ not applicable).

Two RT studies [58,89] reported the percentage of type I fibers, with small and statistically non-significant pooled effect (MD = 0.14% (−1.38–1.66%); *p* = 0.86; I^2^ = 0%). The same two studies reported the percentage of type IIa fibers, showing slight but statistical non-significant increase (MD = 1.03% (−0.43–2.48%); *p* = 0.17; I^2^ = 11%). Finally, one RT study [58] reported a statistically significant increase in the percentage of type IIx fibers (MD = 2.42% (1.96–2.88); *p* < 0.001; I^2^ not applicable).

### 3.6. Effects of RT and WBV on Body Functional Performance

**Figure 6:**
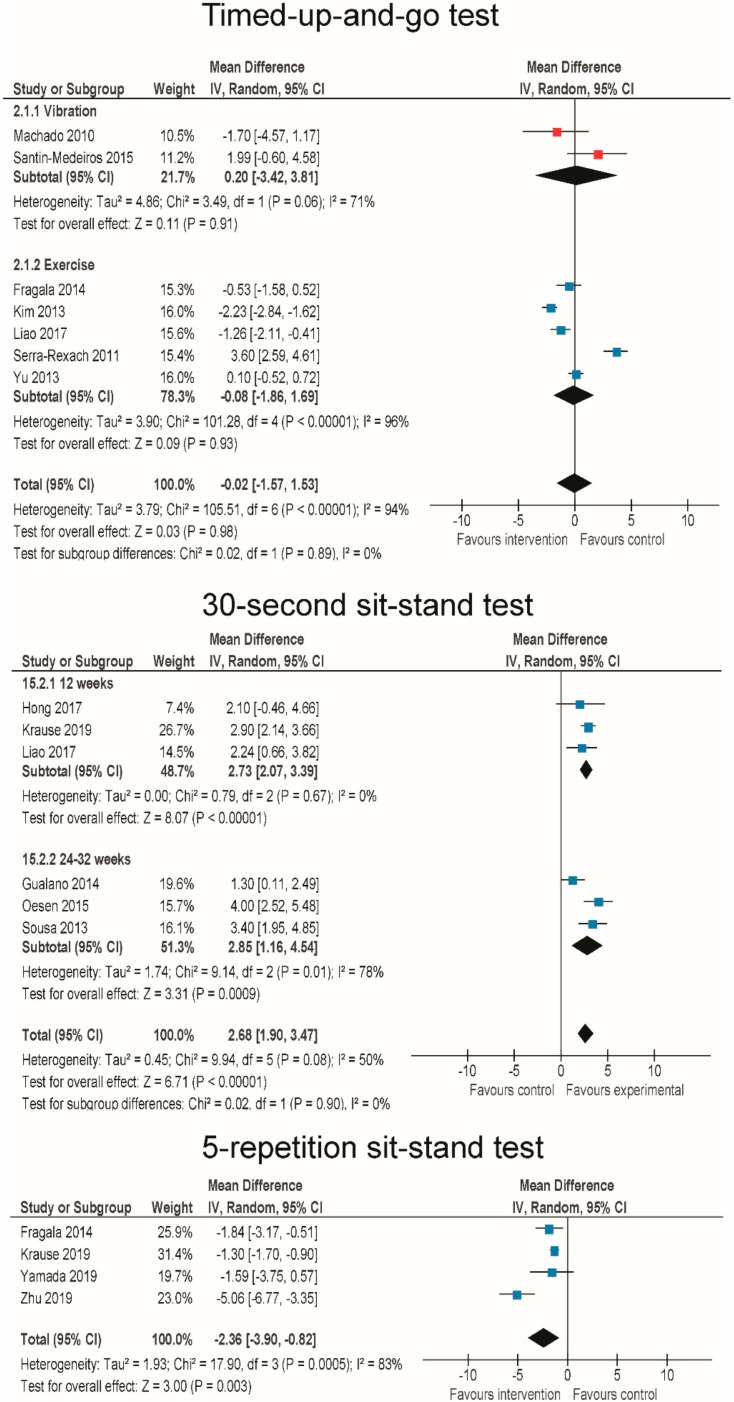
Effects of whole-body vibration and resistance exercise on functional mobility tests.

The results on functional performance are summarized in Figure 6. The timed up-and-go test was performed in 2 WBV [87,88] and 6 RT studies [52,55,69,75,90]. Overall, there were no differences between intervention and control groups across all studies (MD = −0.12 s (−1.36–1.12 s); *p* = 0.85; I^2^ = 93%). There were also no differences between the WBV and RT (MD = 0.20 and −0.08 s, respectively; *p* = 0.89 for subgroup differences). The 30-s sit-stand test was performed in 6 RT studies [55,59,68,76,80,85], with an overall improvement of 2.68 repetitions (1.90–3.47 repetitions, *p* < 0.001; I^2^ = 0.50%). There was no difference between interventions performed 2 times per week (MD = 2.85 (1.16–4.54 repetitions)) and 3 times per week (MD = 2.73 (2.07–3.39 repetitions)) (*p* = 0.90 for subgroup differences). The 5-repetition sit-stand test was recorded in 4 RT studies [65,67,75,76], and there was a significant improvement (i.e., decreased time to complete the test) across all studies (MD = −2.36 s (−3.9–−0.82 s); *p* = 0.003; I^2^ = 83%).

## 4. Discussion

The purpose of this systematic review with meta-analysis was to investigate the effects of RT, WBV, and EMS interventions on muscle strength, body composition, and functional performance in older adults. It included randomized controlled trials involving at least one intervention group (RT, EMS, or WBV) and a control group were included. In total, 64 studies were included in the meta-analysis (48 RT studies, 12 WBV studies, and 4 EMS studies). The main findings of the present systematic reviews are: (1) knee extension strength was improved by RT and WBV, but not ES; (2) the remaining strength outcomes were only assessed in RT studies and significant positive effects were observed; (3) the effects of RT on body composition were small, while the effects of WBV and EMS are unclear due to the small number of studies; (4) there were small effects on sarcopenia index, while quadriceps cross-sectional area was improved in RT studies, but not WBV studies; (5) functional performance was improved by RT interventions, though not in all tests. Overall, the RT interventions proved to be effective for improving muscle strength, muscle cross-sectional area and functional performance, while the effects on body composition were small or non-existent. WBV seems to be comparably effective for improving muscle strength, but not muscle cross-sectional area. A major limitation of the review is the smaller number of WBV and particularly EMS studies. Comparisons between the different intervention types were therefore limited and were not possible for several outcome measures. Subgroup analyses revealed that some of the independent variables (duration of intervention, weekly frequency, type of resistance in RT studies, and age of participants) might have influenced the results; however, these findings were not statistically significant and cannot be conclusively confirmed.

The positive effects of RT, WBV, and EMS in older adults have been reported numerous times [9,10,11,13,14,19,22,23,25,26,30,31,91,92,93]. In this review, we included only randomized controlled trials that included at least one group that did not receive any interventions (control group). While the positive effects of RT were clearly demonstrated, the effects of WBV, and in particular EMS, were smaller or absent. Individual studies that directly compared RT and WBV have shown similar effects of the two interventions related to muscle strength and power outcomes [25,26]. In a non-controlled single-group study, improvements in muscle strength and power and functional performance were also observed after 9 weeks of WBV [94]. While the present review showed improvements in muscle strength after WBV interventions, only 2 WBV studies that assessed functional performance were included. Therefore, the effects of WBV on functional performance remain unclear. Since improvements in functional performance are often observed in parallel with increases in muscle strength [92,95,96] and muscle power [97], it can be expected that WBV will also increase functional performance. In addition to increases on muscle strength and possible improvements in functional performance after WBV, previous research also showed positive effects of WBV on postural balance [22], cardiovascular outcomes [98] and possibly muscle activation [99] in older adults. Overall, we can recommend the prescription of WBV to older adults, but it cannot be guaranteed that WBV will produce comparable effects to RT in view of all outcomes relevant to health and well-being.

EMS has been used extensively in people who cannot engage in normal physical activity and has been shown to produce somewhat similar responses to exercise at the muscular level [100]. In this review, a very limited amount of randomized controlled trials has been identified to investigate the effects of EMS in older adults. Our analyses could not confirm or indicate any effects of EMS interventions. EMS has previously been shown to be effective in counteracting muscle weakness in advanced disease [101] and sarcopenia in older adults [30,32], and even to provide additive effects in terms of morphological outcomes when combined with RT in healthy adults [102]. However, the effect of EMS on functional performance of the older adults are less consistent [103]. Nevertheless, the above-mentioned promising results should be re-evaluated in randomized controlled trials to strengthen the findings and enable better comparison to RT and WBV. Based on the results of this and previous research [92], the use of EMS should be encouraged when performing physical activities is not possible or older adults are not motivated to perform it.

Across all interventions, the improvements in muscle strength were much more evident than improvements in muscle mass. It is known that improvements in strength due to neural adaptations occur much earlier before a meaningful increase in muscle mass is seen [104]. While most of the interventions in the present review lasted 12 weeks or longer, improvements in muscle mass could nonetheless be expected. It is possible that muscle mass measurements are not reliable enough to detect the effect of the interventions. Alternatively, the cross-sectional area of the quadriceps was statistically significantly increased across RT studies in this review. Moreover, a previous review also reported notable increases in the cross-sectional area of thigh muscles (+2.31 cm^2^) in older adults aged >75 years [105]. Interestingly, the latter review reported such effects for WBV, while the pooled effects of the WBV studies in our review were small.

The results on functional performance were different across outcome measures. Neither WBV, RT nor EMS improved the performance of the timed up-and-go test. Conversely, the sit-stand performance was significantly improved by RT interventions (an increase of 2.68 repetitions in 30-s sit-stand task and a decrease of 2.36 s in the 5-repetition sit-stand task time). It should be noted that the results regarding functional performance were significantly influenced by the heterogeneity of the studies. In particular, the timed up-and-go test performance was substantially improved (−1.77 s) in one study and reduced even more in the second study (+1.99 s). Similarly, most RT studies showed improvements in this test, but one study [69] showed a large reduction (+3.6 s), which led to a negligible pooled effect. This particular study was conducted on very old participants (> 90 years) and included a short-term resistance exercise program, based on light to moderate loads. If this study is excluded from the analysis, the pooled effect size would show statistically significant positive improvements (MD = −0.93 s; *p* < 0.001).

The secondary aim of this paper was to determine the independent variables, related to the interventions, that can influence the magnitude of the outcomes. Most of the subgroup analyses that could be conducted as the number of studies was sufficient, showed no such statistically significant effects. There were statistically non-significant trends for lower limb muscle mass and leg press strength to be improved more with a higher (≥3) weekly session frequency. The literature in the field of sports science [106,107] suggests that weekly frequency is not an independent factor for improvements in muscle strength and muscle mass. A recent meta-analysis suggested that similar is true for older adults [108], although a minimum of 2 sessions per week is typically recommended. Our results also indicated a potentially higher effect of interventions based on machine training and free weights, compared to elastic resistance and approaches combining several types of resistance. In the general population, the effect of elastic resistance appears to be essentially the same as machine-based resistance and free weights [109]. Note that our observation on lesser effects of elastic resistance compared to machines and free weights is limited to knee extension strength and that the difference between the effect of elastic resistance (SMD = 0.91) and machine-based resistance (SMD = 1.36) and free weights (SMD = 1.33) was not statistically significant (*p* = 0.68). Therefore, it is probably appropriate to include elastic resistance in RT programs for older adults.

The first limitation of this systematic review with meta-analysis is the inclusion of only randomized controlled trials. While this was done to compile only high-quality evidence, important findings from studies with different designs were omitted. In particular, the number of EMS studies was very small. It should be emphasized that the lack of reported effects of EMS in the review is partly due to the lack of randomized controlled trials and not necessarily because the EMS is not effective. Furthermore, a major limitation of the review is the high heterogeneity of the studies, which precluded more subgroup analyses and is potentially a major confounding factor. Partially, we investigated this issue with several sensitivity analyses which showed that the results were not heavily influenced by certain factors, such as type of measurements (for knee strength), presence of sarcopenia (though somewhat smaller effects were observed in elderly sarcopenia patients), and adherence to studies. Because there are several factors that can influence response to resistance exercise (in particular, the characteristics of the intervention in addition to those mentioned above), we did our best to perform subgroup analyses to exclude or confirm several factors, such as exercise frequency, intervention duration, and resistance exercise type. Nevertheless, some of the variability between the interventions could not be accounted for. Therefore, we strongly emphasize that these results should be viewed with high caution. Future studies and practitioners should not use the numbers we obtained as a standalone guideline, but rather view our analyses as an exploration of general trends in the field of interventions for older adults.

## 5. Conclusions

This paper reviewed RCT studies that examined the effects of RT, WBV, and EMS on muscle strength, body composition, and functional performance of older adults. It was found that RT and WBV are effective for increasing muscle strength, while the data was very limited for EMS. RT interventions also improve functional performance and increase muscle-cross sectional area but have no effect on muscle mass. Further studies exploring the effect of WBV and in particular of EMS are needed for better comparison with RT. For the time being, EMS can be recommended for people that are unable to perform RT or WBV. Otherwise, RT or a combination of RT and WBV or EMS is probably the most efficient way to improve muscle strength and functional performance, while the best approach to increase muscle mass in older adults still needs to be determined by further studies. Due to the several limitations of this review, we urge the readers to view the results with caution.

## Supplementary Materials

The following are available online at https://www.mdpi.com/2077-0383/9/9/2902/s1, Supplementary data 1: Detailed data regarding study outcomes, interventions and participants.
